# Shock Synthesis of Decagonal Quasicrystals

**DOI:** 10.1038/s41598-017-15229-4

**Published:** 2017-11-15

**Authors:** J. Oppenheim, C. Ma, J. Hu, L. Bindi, P. J. Steinhardt, P. D. Asimow

**Affiliations:** 1 Division of Geological and Planetary Sciences, California Institute of Technology, 1200 E. California Blvd. M/C170-25, Pasadena, CA 91125 USA; 2 Dipartimento di Scienze della Terra, Università degli Studi di Firenze, Via La Pira 4, I-50121 Firenze, Italy; 3 CNR-Istituto di Geoscienze e Georisorse, Sezione di Firenze, Via La Pira 4, I-50121 Firenze, Italy; 4 Department of Physics, Princeton University, Jadwin Hall, Princeton, NJ 08544 USA; 5 Princeton Center for Theoretical Science, Princeton University, Princeton, NJ 08544 USA

## Abstract

The Khatyrka meteorite contains both icosahedral and decagonal quasicrystals. In our previous studies, icosahedral quasicrystals have been synthesized and recovered from shock experiments at the interface between CuAl_5_ and stainless steel 304 alloys. In this study, we report a new shock recovery experiment aimed at synthesizing decagonal quasicrystals similar to decagonite, natural Al_71_Ni_24_Fe_5_. Aluminum 2024 and permalloy 80 alloys were stacked together and shocked in a stainless steel 304 recovery chamber. Abundant decagonal quasicrystals of average composition Al_73_Ni_19_Fe_4_Cu_2_Mg_0.6_Mo_0.4_Mn_0.3_ with traces of Si and Cr were found along the recovered interface between the Al and permalloy. The experiment also synthesized AlNiFe alloy with the B2 (CsCl-type) structure and the metastable Al_9_Ni_2_ phase. We present chemical (scanning electron microscopy and electron microprobe) and structural (electron backscatter diffraction and transmission electron microscopy) characterization of the recovered phases and discuss the implications of this shock synthesis for the stability of quasicrystals during high-pressure shocks and for the interpretation of the phase assemblage found in Khatyrka.

## Introduction

Unlike periodic crystals, which have 1-, 2-, 3-, 4-, or 6-fold rotational symmetries, quasicrystals are aperiodic structures^1,2^ that can exhibit previously forbidden rotational symmetries such as 5-, 8-, 10-, and 12-fold. Three types of natural quasicrystal have been discovered in (and, so far, *only* in) the Khatyrka meteorite^3–7^. Two of these, Al_63_Cu_24_Fe_13_ icosahedrite (all formulas herein are on an atomic basis) and Al_62_Cu_31_Fe_7_ i-Phase II, have icosahedral symmetry, featuring six five-fold axes of rotational symmetry^4^. On the other hand, Al_71_Ni_24_Fe_5_ decagonite displays a single 10-fold rotational symmetry axis together with periodic patterns taken perpendicular to the 10-fold direction^5^. The occurrence of natural quasicrystals in a meteorite demonstrating evidence of a high-pressure shock and rapid post-shock cooling^8^ suggests that the passage of a shock wave facilitates nucleation and growth of quasicrystals and perhaps relaxes the constraints on precise ratios of starting materials required by static synthesis methods. We have experimentally confirmed this idea in previous reports of successful laboratory shock synthesis of icosahedral quasicrystals analogous to icosahedrite but with higher Al contents and five significant components (Al, Cu, Fe, Cr, Ni)^9,10^.

The quasicrystals in Khatyrka coexist with a number of other exotic, but crystalline, metallic phases, including aluminum (Al), stolperite (CuAl), kryachkoite (Al,Cu)_6_(Fe,Cu), hollisterite (Al_3_Fe)^11^, khatyrkite (CuAl_2_), cupalite (CuAl), and steinhardtite^12^. Steinhardtite is a new polymorph of Al and exhibits a body-centered cubic (bcc) structure, unlike the common phase of aluminum, which has a face-centered cubic (fcc) structure.

In this study, we used an experimental technique similar to Asimow *et al*.^9^ to try to synthesize decagonal quasicrystals (d-QC) similar to decagonite^6^ in order to further confirm the hypothesis that natural quasicrystals in the Khatyrka meteorite are explained by shock processing of unusual metallic alloys during collisions in the asteroid belt. Because the goal of this work is primarily to show that synthesis of Al-Ni-Fe d-QC by shock is straightforward, we did not go to extraordinary ends to match the starting materials to those present in Khatyrka. Instead we selected readily available commercial alloys. An aluminum 2024 disc was loaded into a permalloy 80 cup within a stainless steel 304 (SS304) recovery chamber. The target was impacted by a Ta flyer at 1041 ± 1 m/s and then the chamber was recovered intact, sawn open, polished, and examined by scanning electron microscopy (SEM) in imaging, energy dispersive X-ray spectroscopy (EDS), and electron backscatter diffraction (EBSD) modes; by wavelength-dispersive electron probe microanalysis (EPMA); and by transmission electron microscopy (TEM) on a section extracted by Focused Ion Beam (FIB) milling, in bright-field imaging, scanning, EDS, and selected-area electron diffraction (SAED) modes.

## Results

### SEM and EPMA analysis

**Figure 1:**
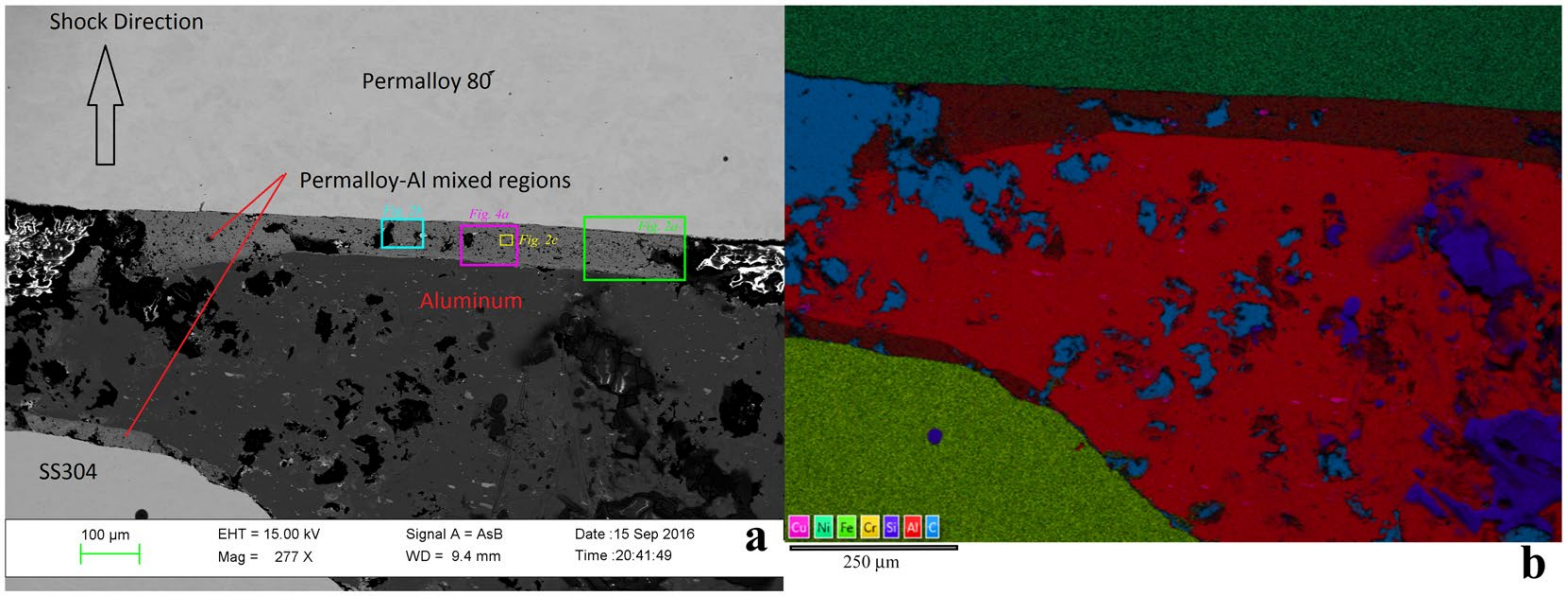
General views of the polished surface of recovered specimen from shot S1235. First shock propagated from bottom of images towards top. (**a**) Backscattered electron image, showing the initially 1 mm thick Al2024 layer attenuated by flow associated with impact deformation to ~0.5 mm in this area near the left side-wall of the chamber. Regions of intermediate backscatter contrast mark a reaction zone up to ~0.1 mm wide with mean atomic number intermediate between the Al-rich and Ni-rich starting materials. (**b**) X-ray intensity map of the same general area, using the color scheme shown at lower left. Voids in the sample appear cyan due to Carbon in the epoxy fill. The brown regions mark the mixed layers.

Figure 1 displays a general view of the polished surface of the recovered sample chamber, extending across the full width of the Al2024 layer, attenuated by the cratering flow to about half its original thickness. In both backscattered electron contrast images and false-color EDS X-ray maps, the unreacted SS304, Al2024, and permalloy 80 regions are evident along with newly-formed reacted zones incorporating elements from both the Al2024 and permalloy 80 starting materials. The SS304 does not appear to have participated extensively in any reactions. The thickest observed mixed layer, about 100 μm wide, extends across the full rear (down-range) contact between the starting materials. The thinner layer that appears, in this section, to occupy the boundary between the SS304 and Al2024 is also formed by mixing Al2024 and permalloy 80, derived from the side-wall of the permalloy cup around the Al2024.

**Figure 2:**
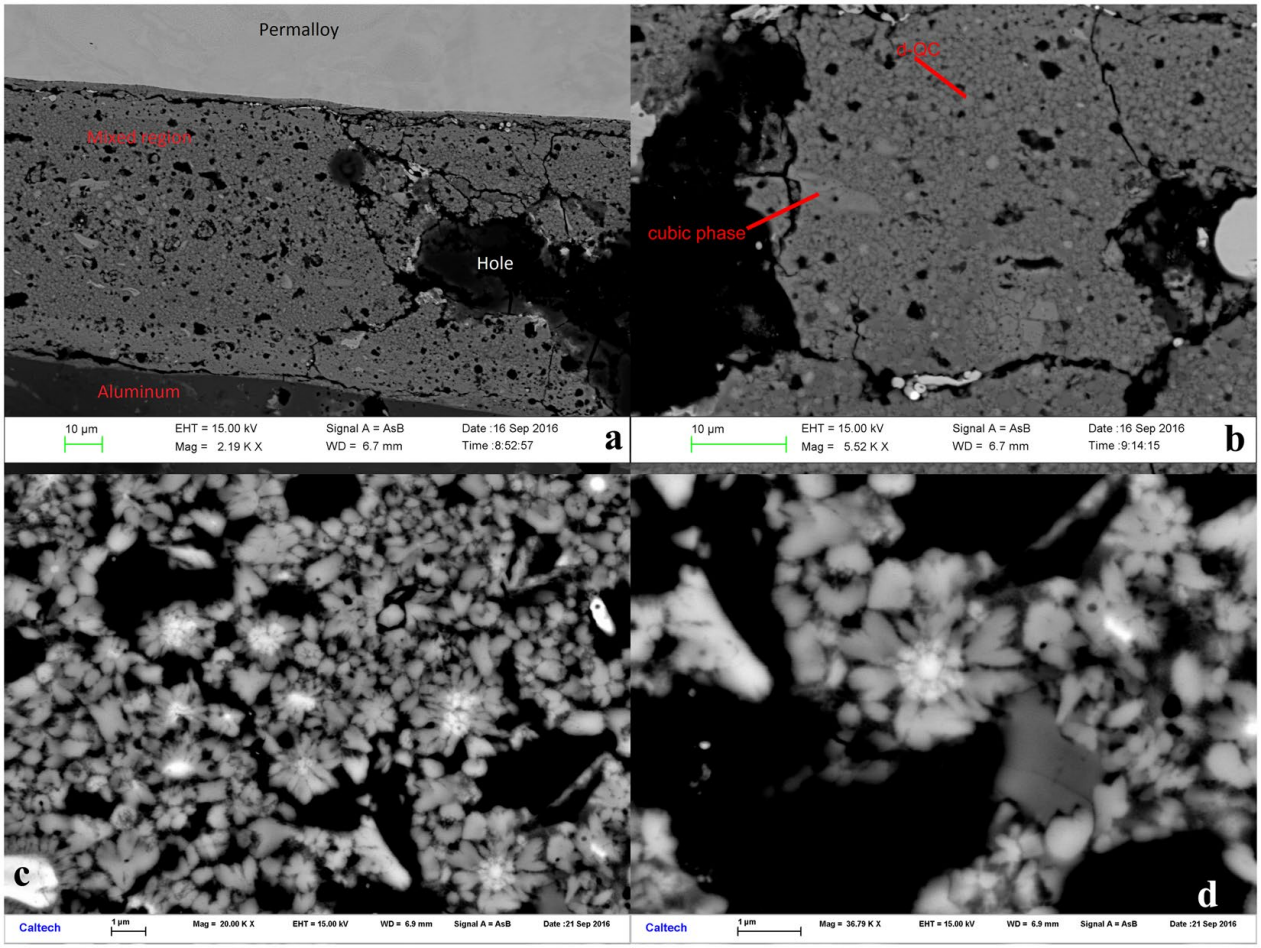
Progressive enlargement of backscattered electron images of the mixed layer (See Fig. 1a for locations of frames): (**a**) Within the ~100 μm wide mixed layer, porosity and 1–10 μm grains of distinct backscatter contrast are visible. (**b**) Further enlargement shows angular ~10 μm grains of a cubic phase and rounded ~1 μm grains identified as “d-QC”. (**c**) The dark spots are voids, the dark gray grains are Al_9_Ni_2_, the light gray are d-QC, and the bright white areas are the cubic phase. (**d**) A “flower” with a core of cubic phase and petals of d-QC radiating outward. Many of the flowers appear to display 10-fold symmetry.

Viewing the rear contact mixed layer at progressively higher magnification in Fig. 2, it becomes clear that this region contains mostly ~1 μm sized rounded grains with a few ~10 μm angular grains and numerous voids ranging from 1 to 100 μm across. It is not clear whether these are representative of actual voids in the volume of the recovered sample, formed upon decompression from the shock state, or whether they are only surface features formed by plucking during polishing. However, the holes exposed on the surface were filled with epoxy in order to allow better polishing of the surrounding metallic areas.

**Figure 3:**
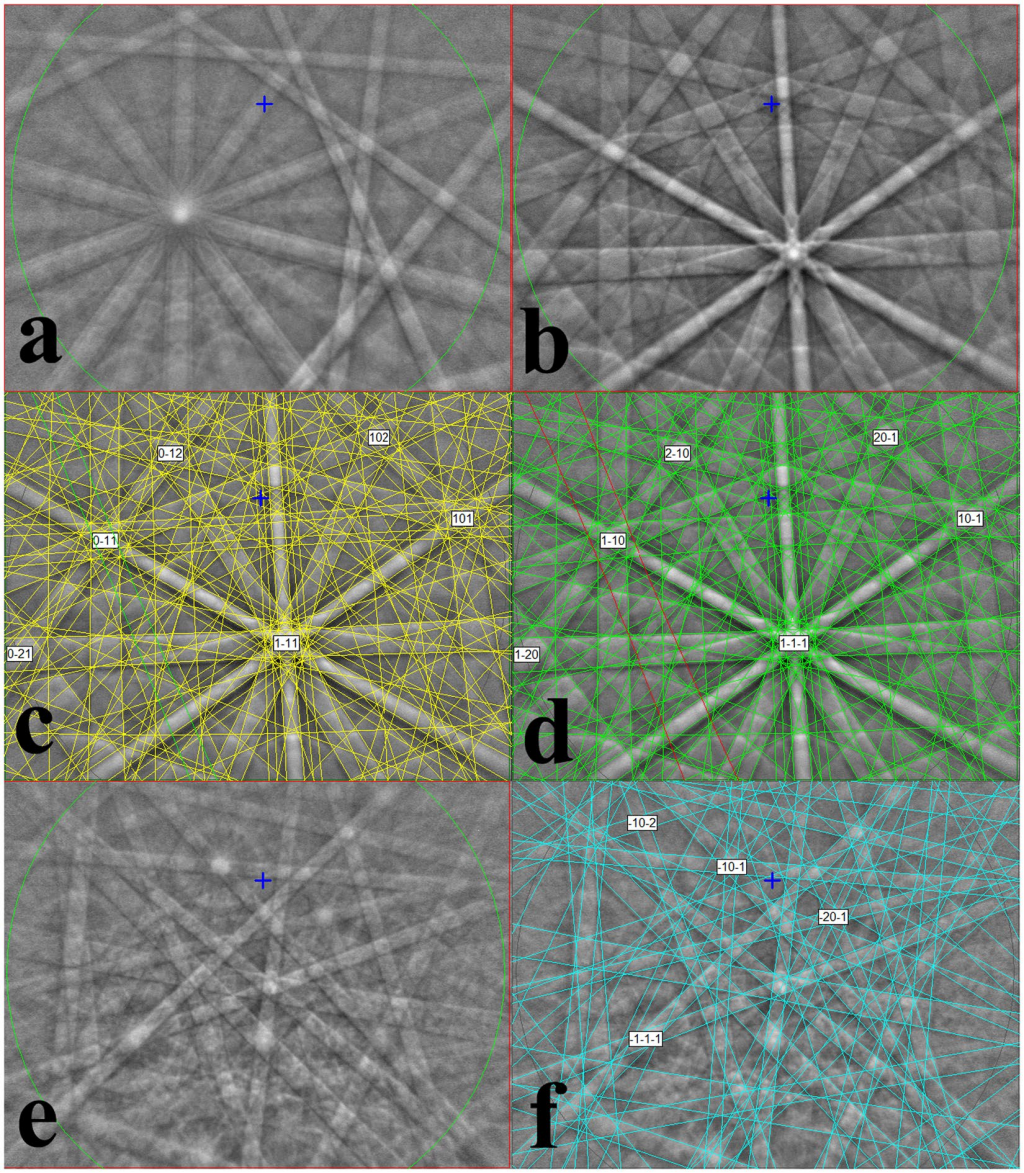
Kikuchi patterns from Electron Backscatter Diffraction of selected phases in S1235. (**a**) d-QC displaying the 10-fold zone axis. The pattern is clearly distinct from the 5-fold zone axes observed in icosahedral quasicrystals by Asimow *et al*.^9^. (**b**) Unindexed pattern of cubic phase. Note the faint superposed double-band running from upper-left to lower-center. (**c**) Indexing of pattern from (**b**) as *Pm* $\bar{3}$ *m* B2 (CsCl) structured cubic phase, with the green band representing the (111) plane highlighted as the narrow member of the double-band. (**d**) Attempt to index pattern from (**b**) as *Im* $\bar{3}$ *m* A2 (bcc) structure, with the red band representing the (222) plane; note that (111) is forbidden in this structure. Hence the EBSD result favors the B2 structure for this phase. (**e**) EBSD pattern of Al_9_Ni_2_ phase. (**f**) Indexing of pattern (**e**) as monoclinic space group 14.

Phase identification of the various materials delineated by distinct backscatter contrast is achieved with point EDS analyses and EBSD patterns to measure composition and structure at common points. The light gray, typically micron-sized, often rounded grains display Kikuchi patterns with characteristic 10-fold symmetry (Fig. 3a) uniquely associated with decagonal quasicrystals. As expected, since the d-QC structure has only one 10-fold symmetry axis, a relatively small fraction of grains show the 10-fold zone axis within the cone sampled by the EBSD detector (in fact, the d-QC have a preferred orientation, see below). EDS analysis of these points shows that they contain, above detection limit, observable concentrations of seven metals: Al, Ni, Fe, Cu, Mg, Mo, and Mn. Quantitative analysis by electron microprobe shows an average composition for these regions, expressed on a 100-atom basis, Al_73.3_Ni_19.3_Fe_4.3_Cu_1.8_Mg_0.6_Mo_0.4_Mn_0.3_ (see Table 1 for ranges in composition based on standard deviation among 8 analytical points).

**Table 1:** Electron Probe analysis of S1235.

	d-QC	B2 cubic phase 1	B2 cubic phase 2	Al_9_Ni_2_
n	8	6	6	5
Weight percent				
Al	56.2 ± 0.7	45.3 ± 0.6	31.0 ± 0.5	63.9 ± 0.6
Fe	6.8 ± 0.5	6.8 ± 0.5	9.6 ± 0.5	6.1 ± 0.4
Cu	3.2 ± 0.6	3.3 ± 0.5	3.0 ± 0.5	1.5 ± 0.4
Cr	*nd*	*nd*	0.16 ± 0.08	*nd*
Ni	32.2 ± 1.1	44.2 ± 1.1	49.9 ± 1.1	23.0 ± 0.7
Mo	1.0 ± 0.3	1.3 ± 0.3	2.9 ± 0.3	0.9 ± 0.2
Mn	0.4 ± 0.3	0.4 ± 0.3	0.6 ± 0.3	0.5 ± 0.3
Mg	0.4 ± 0.1	0.3 ± 0.1	0.3 ± 0.1	0.40 ± 0.04
Si	*nd*	*nd*	0.14 ± 0.06	*nd*
Total	100.3	101.7	97.4	96.3
Normalized atomic percent				
Al	73.3 ± 0.9	63.5 ± 0.8	50.4 ± 0.8	80.8 ± 0.7
Fe	4.3 ± 0.3	4.7 ± 0.3	7.6 ± 0.4	3.8 ± 0.3
Cu	1.8 ± 0.3	2.0 ± 0.3	2.1 ± 0.3	0.8 ± 0.2
Cr	*nd*	*nd*	0.14 ± 0.06	*nd*
Ni	19.3 ± 0.7	28.5 ± 0.7	37.4 ± 0.9	13.4 ± 0.4
Mo	0.4 ± 0.1	0.5 ± 0.1	1.3 ± 0.1	0.3 ± 0.1
Mn	0.3 ± 0.2	0.2 ± 0.2	0.4 ± 0.2	0.3 ± 0.2
Mg	0.6 ± 0.1	0.4 ± 0.1	0.5 ± 0.1	0.56 ± 0.06
Si	*nd*	*nd*	0.22 ± 0.10	*nd*

Several other phases were identified in intimate contact with the d-QC in the mixed region. First, a cubic structure (Fig. 3b) was found, with two distinct groups of compositions. The average analyses of the two compositions are Al_63_Ni_29_Fe_5_Cu_2_Mo_1_ and Al_50_Ni_37_Fe_8_Cu_2_Mo_1_ (Table 1; the sums of these formulas may differ from 100 due to rounding and minor components). The EBSD patterns of two candidate structures for this phase, the *Im* $\bar{3}$ *m* (A2 or bcc) structure (like steinhardtite) and a *Pm* $\bar{3}$ *m* primitive cubic unit cell structure (B2 or CsCl-type structure), are quite similar. The phase diagram of the Al-Ni-Fe system^13^ suggests that both compositions plot in the stability field of the B2 phase. Careful examination of the EBSD patterns shows that the (111) reflection is present (Fig. 3c), which is a forbidden reflection in bcc (Fig. 3d) and indicates that the phase is B2. This result was confirmed by TEM study, see below. We also observe an Al_9_Ni_2_ phase with monoclinic *P*2_1_/*c* Al_9_Co_2_-type structure and typical composition Al_81_Ni_13_Fe_4_Cu_1_Mg_1_ (Fig. 3e,f and Table 1). Al_9_Ni_2_ is a metastable alloy that only forms during rapid solidification^14^.

The cubic phase and the d-QC are closely associated and often form a core-and-petal flower texture (Fig. 2d). Oddly, the flowers appear to have an overall 10-fold symmetry in many sections, even though the phase at the core of the flower is cubic and lacks such a symmetry element. This pattern resembles in some ways the texture of crystal clusters observed in synthetic Zn-Al-Cr alloys^15^, where multi-twins sharing a five-fold symmetry axis led to the suggestion that crystals nucleated on a short-lived icosahedral cluster or nucleus. We speculate that the cubic phase at the center of the core-and-petal structures in our experiment may be recrystallized from an initial transient phase with decagonal symmetry, but this idea requires further investigation.

### TEM analysis

**Figure 4:**
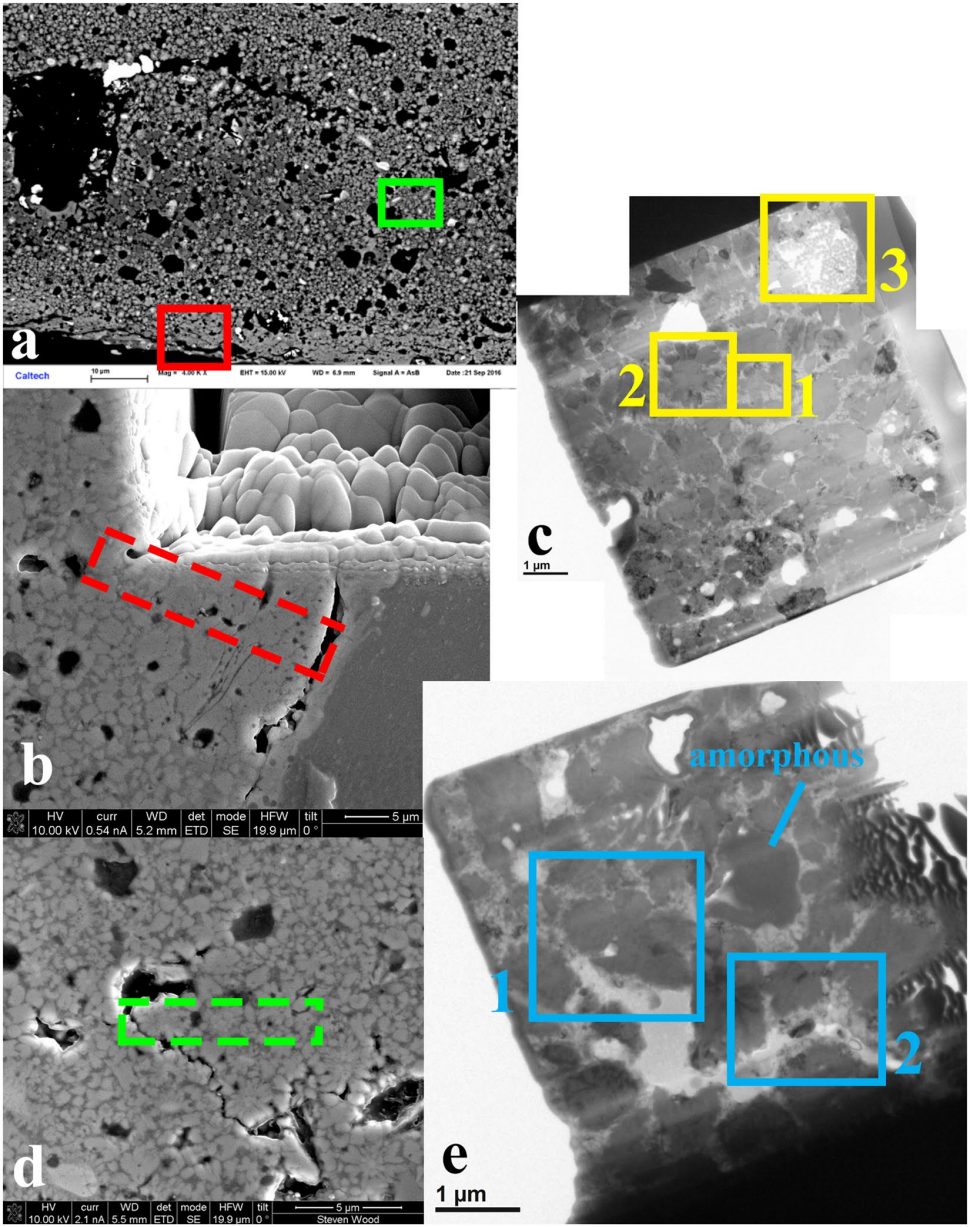
Extraction and preparation of FIB sections for TEM analysis. (**a**) Backscattered electron image showing variation in texture across the mixed region. FIB section A was extracted from the denser-textured area (red box) close to the Al2024 layer. FIB section B was taken from an area with two prominent “flowers” (green box). (**b**) Secondary electron image of the area targeted for FIB section A liftout, shown by the dashed red rectangle. (**c**) Bright-field TEM image of extracted and thinned FIB section A. Regions of Interest 1, 2, and 3 are further imaged and described below (Figs 5, 6, 7 and 8). (**d**) Secondary electron image of the area targeted for FIB section B liftout, shown by the dashed green rectangle. (**e**) Bright-field TEM image of extracted and thinned FIB section B. Regions of Interest 1 and 2 are further imaged and described below (Figs 9 and 10) and the region labeled “amorphous” is discussed in the text.

Two FIB sections were extracted from the reaction boundary area between the Al-alloy 2024 and permalloy (Fig. 1). The reaction region has two distinct textures (Fig. 4a). The first texture consists of coarse grains of d-QC, the cubic AlNiFe phase, and Al_9_Ni_2_, with significant porosity. The other texture, closer to the Al2024 side of the reaction zone, is denser in appearance and includes predominantly fine-grained d-QC. FIB section A was extracted from the second textural area, because we presumed it would be more coherent during milling and liftout. The extracted and thinned FIB section A, taken from the area shown in a higher-magnification secondary electron image in Fig. 4b, is shown in a bright-field TEM image in Fig. 4c, with three selected regions of interest highlighted. FIB section B was later taken from the first textural area, displaying better-formed “flowers”, and shown in secondary electron image Fig. 4d and bright-field TEM image Fig. 4e with two selected regions of interest highlighted. The nearly uniform diffraction contrast of the d-QC grains in FIB section B indicates a shared orientation, with most of their 10-fold axes in the plane of the section and roughly parallel to the direction of shock propagation. One indicated grain in FIB section B is amorphous (Fig. 4e) and has high gallium content, inferred to be from Ga beam damage amorphization during sample preparation.

**Figure 5:**
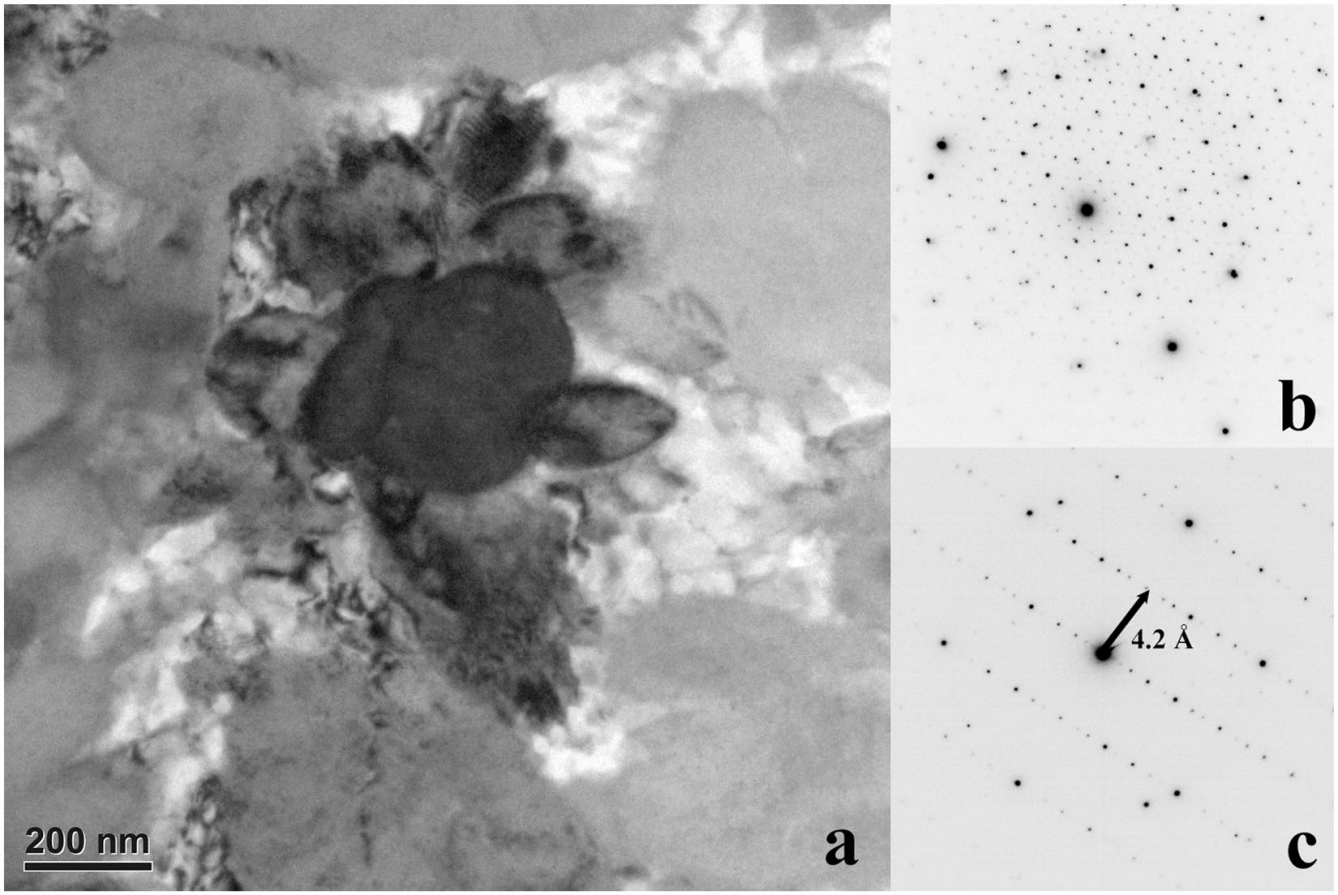
Region of Interest 1 from FIB section A (see Fig. 4). (**a**) Bright-field image of a decagonal quasicrystal (dark) along the 10-fold rotation zone axis. The surrounding fine grains are quasicrystals of different orientations. (**b**) SAED pattern of the quasicrystal along the 10-fold rotation axis. (**c**) SAED pattern perpendicular to the 10-fold rotation axis, showing the interlayer spacing corresponding to 4.2 Å.

*FIB Section A*, *Region of Interest 1*: This region includes an equant d-QC surrounded by irregularly shaped QCs (Fig. 5). SAED indicates the decagonal quasicrystal has a stacked-layer structure. Viewed along a zone axis perpendicular to the 10-fold rotation axis, the measured spacing between layers is ~4.2 Å. This is identical to the result obtained on natural decagonite by single crystal X-ray diffraction, an interlayer pacing of 4.208(2) Å^5^. The composition of this d-QC grain by transmission EDS is roughly Al_66_Ni_19_Fe_4_Cu_x_ (copper is not correctly measured in TEM, which has Cu internal parts and the sample is held on a Cu TEM grid).

**Figure 6:**
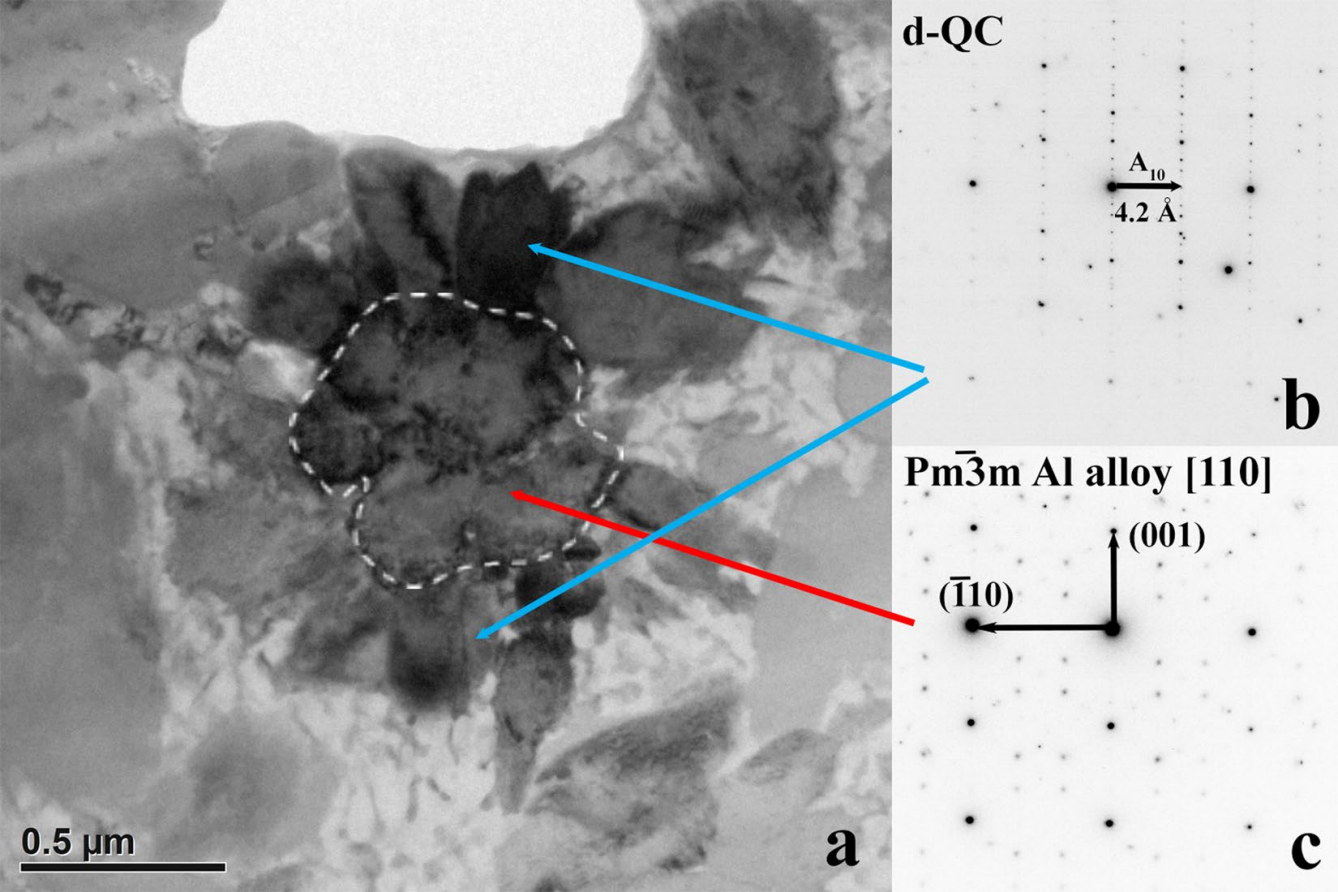
Region of Interest 2 from FIB section A. (**a**) Bright field image. (**b**) In this orientation, two “petals” of dark diffraction contrast display identical SAED images perpendicular to the 10-fold axis of the d-QC. Other petals of weak contrast are d-QC grains along minor zone axes. (**c**) The grain at the center shows the [110] zone-axis orientation of a cubic primitive lattice, with weak spots in the SAED pattern showing superlattice diffraction.

**Figure 7:**
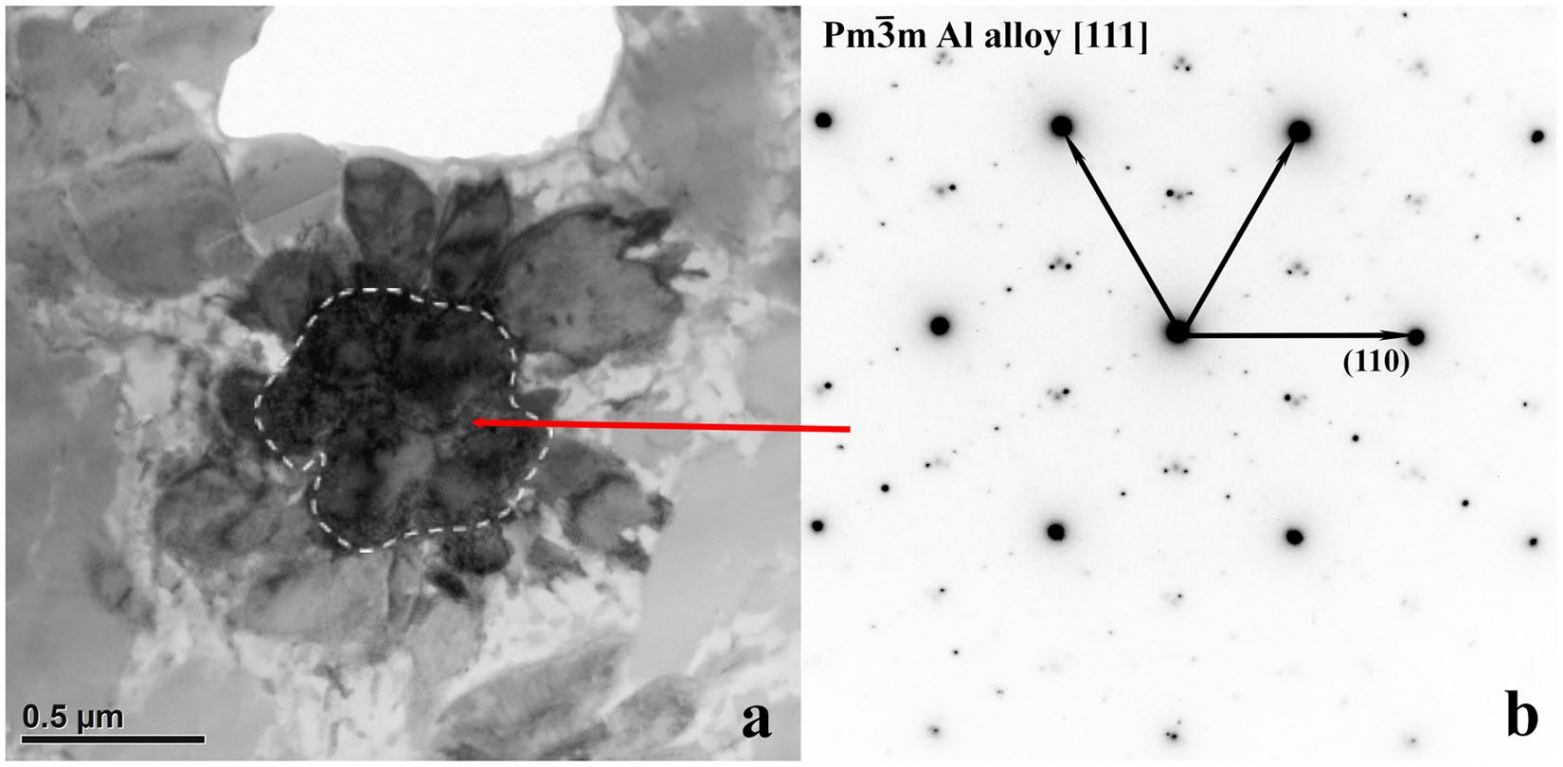
A different orientation of Region of Interest 2 from FIB section A. (**a**) Bright field image; diffraction contrast has changed due to rotation of view relative to Fig. 6a. (**b**) The zone axis of the Al-alloy at the center in this view is [111]. Again, the intermediate weak spots are superlattice diffraction.

*FIB Section A*, *Region of Interest 2*: This region (Figs 6 and 7) contains a flower apparently similar to those observed by SEM. The center phase, shown with moderately dark contrast in the bright field image in Fig. 6 and very dark contrast in Fig. 7, is clearly not bcc-structured steinhardtite. In Fig. 6 it is viewed along the [110] axis and in Fig. 7 along the [111] axis and shows a SAED pattern matching the primitive cubic lattice of a CsCl-type (B2) structure. Its cell parameter is ~3.0 Å. The [110] zone pattern is particularly useful for distinguishing the bcc, face-centered (like α-Al) and primitive cubic lattices (Supplementary Fig. S1).

The composition of the Al-alloy is Al_55_Ni_29_Fe_4_Cu_x_ in Region of Interest 2, comparable to the average of the two cubic structure phases measured by SEM EDS. B2 is the expected structure for Al-rich AlNiFe alloy of this composition^12,13^. In addition, the diffraction pattern shows clear superlattice structure. The weak diffraction spots between the intense spots indicate a large periodicity in the structure (Figs 4 and 5). It could be that the alloy is not perfectly stoichiometric and each unit cell takes less than one vacancy; in such a case, ordering of the vacancies is capable of creating the superlattice structure as shown by the example in Fig. A1 ^16^.

When the B2 Al-alloy grain at the center of the flower is rotated to view along the [110] zone axis, two of the d-QC petals on opposite sides show exactly the same SAED pattern (Fig. 6), indicating the two grains have the same orientation. Moreover, their 10-fold rotation axis is likely parallel to the [001] zone of the Al-alloy. Other quasicrystal petals of Region 2 share different zones at another orientation.

**Figure 8:**
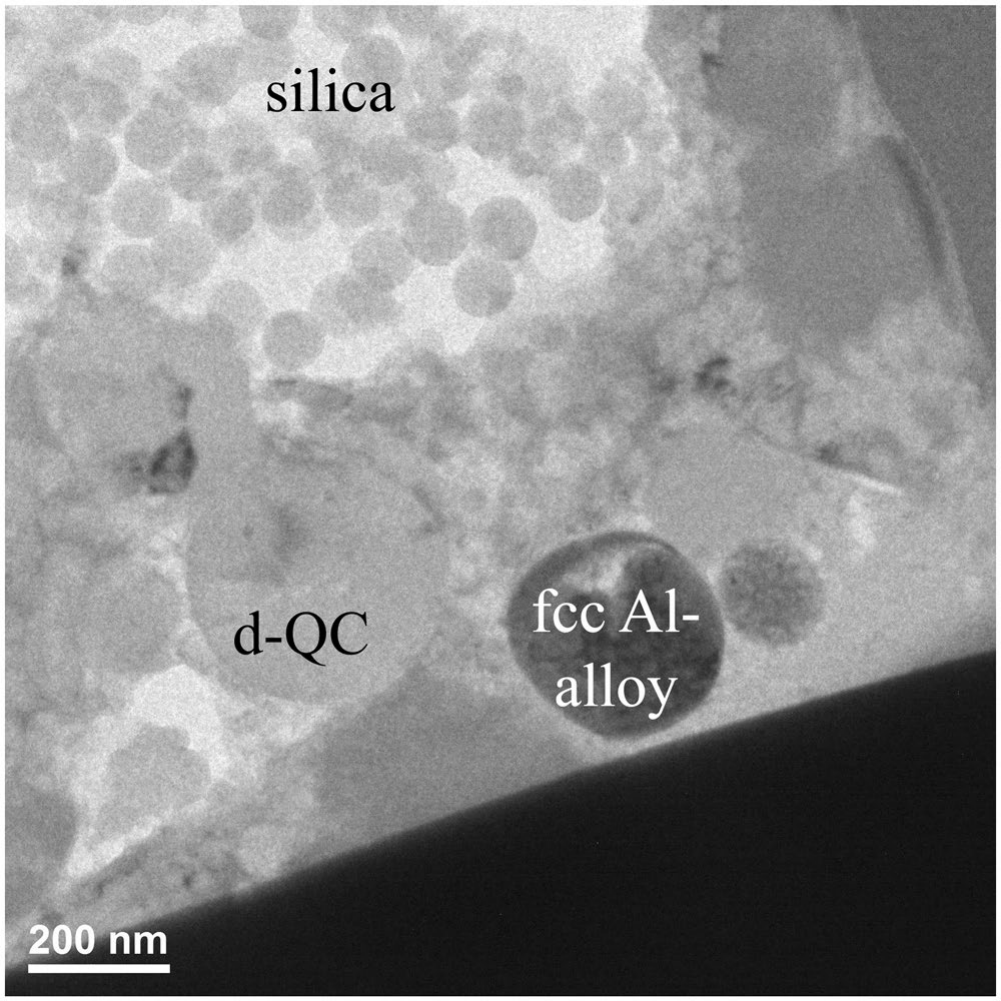
Bright field image of Region of Interest 3 in FIB section A. Details of globules in the area of amorphous silica-rich material are visible.

*FIB Section A*, *Region of Interest 3*: Silica-rich material occurs in the FIB section (Fig. 8). Although some globules appear to have grain boundaries, diffraction patterns collected from the region do not show crystalline material. The silica is likely derived from the colloidal polishing medium used to prepare the section.

**Figure 9:**
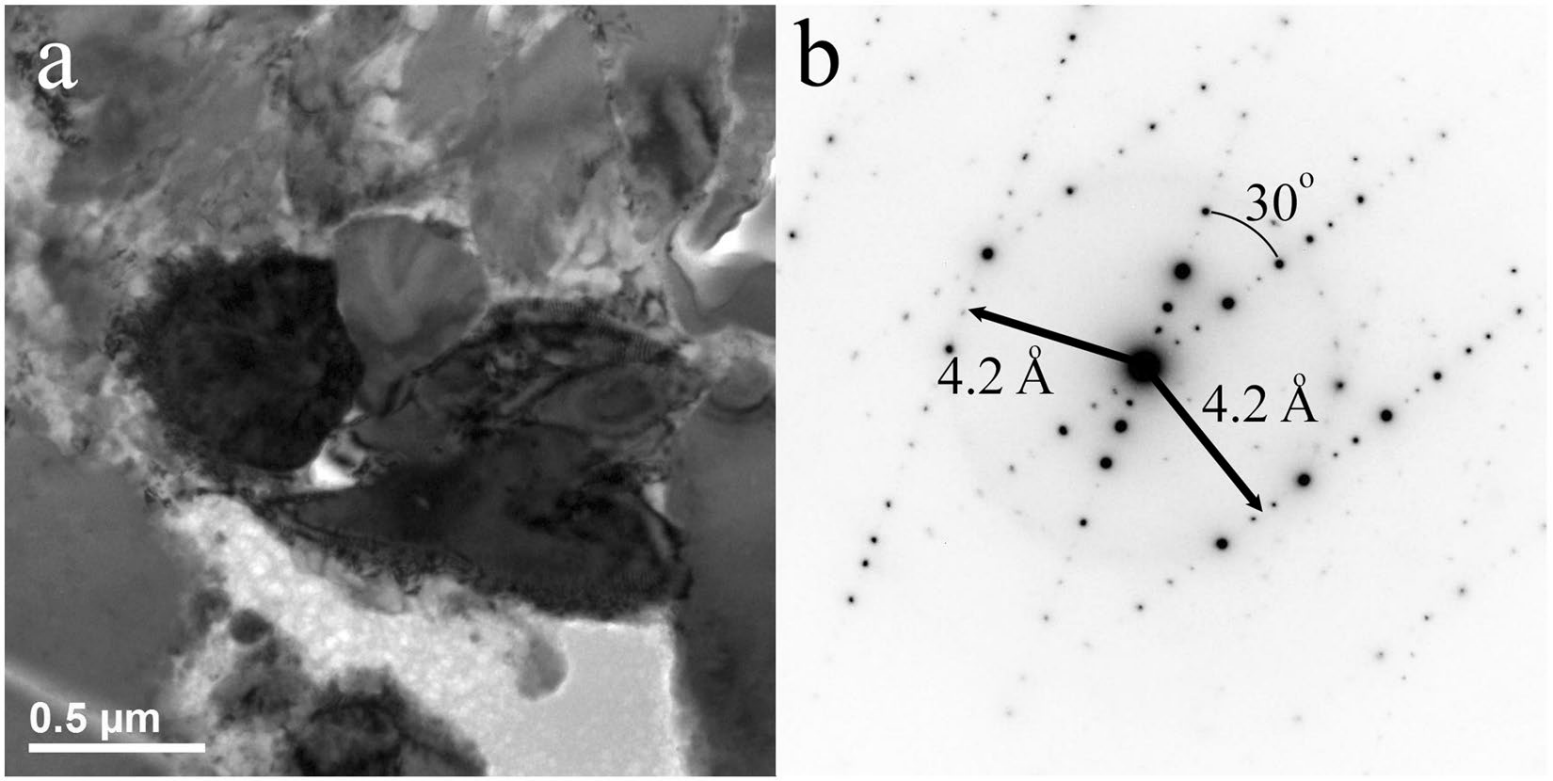
FIB section B, Region of Interest 1. (**a**) Bright-field image of a cluster of d-QC grains showing similar diffraction contrast (darker grey shades). (**b**) Corresponding electron diffraction (SAED) pattern encompassing two of the grains, showing that both are oriented with the 10-fold zone axes in the plane of section and rotated with respect to one another by 30°.

*FIB Section B*, *Region of Interest 1*: This area (Fig. 9) shows a cluster of d-QC grains with their ten-fold axes in the plane of section. SAED including two of the grains shows that both are viewed perpendicular to the 10-fold axis, both have the common 4.2 Å layer spacing, and their 10-fold axes lie at 30° to each other.

**Figure 10:**
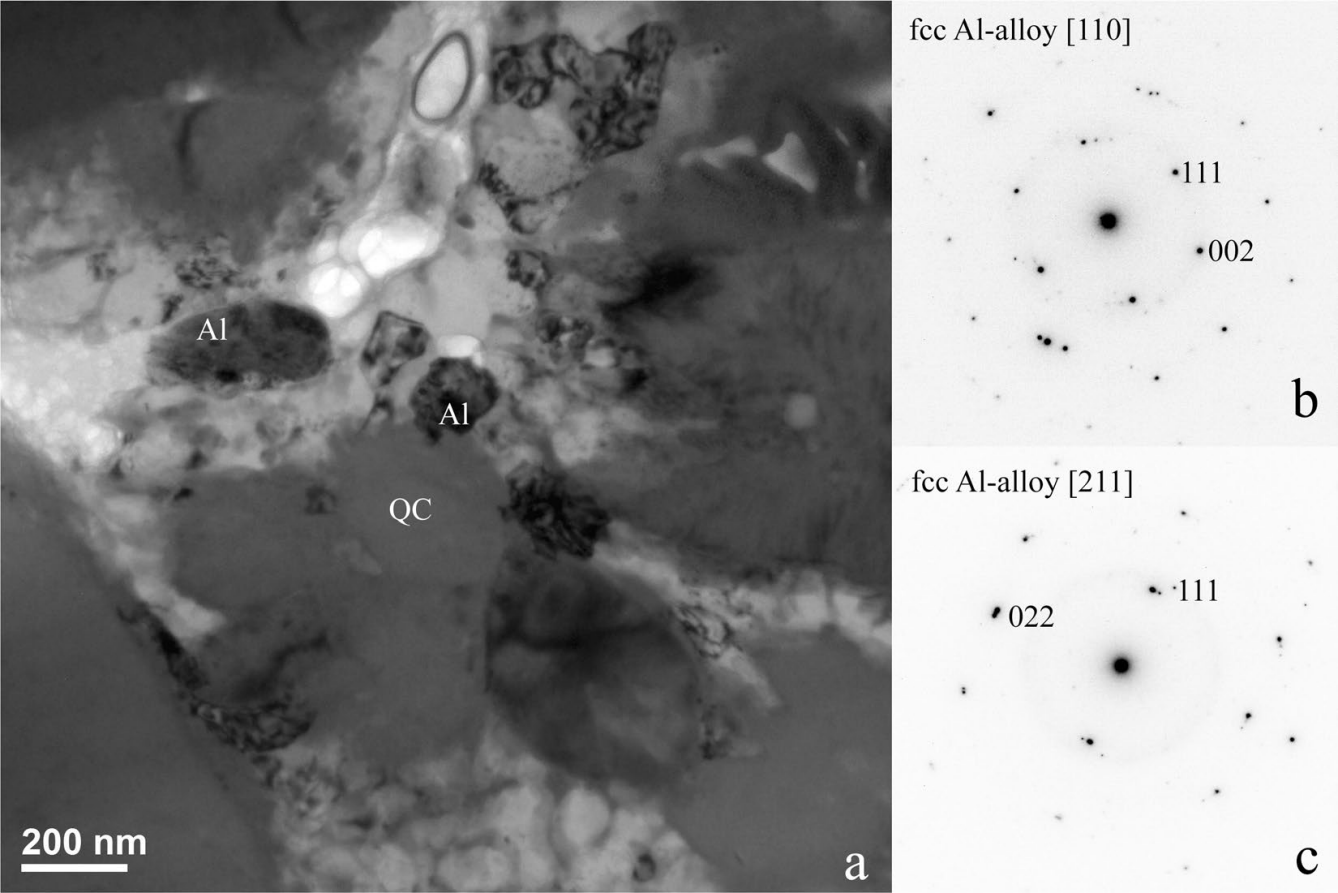
FIB Section B, Region of Interest 2. (**a**) Interstitial aluminum alloy between the QCs has a mottled contrast attributable to local strain and twinning. (**b**) [110] zone of the fcc Al-alloy. (**c**) [211] zone of the fcc Al-alloy. The double spots indicate twinning in the lattice.

*FIB Section B*, *Region of Interest 2*: This area (Fig. 10) contains mottled regions of fcc aluminum interstitial to the d-QC grains. SAED patterns of the fcc Al exhibit doubled spots, consistent with twinning being responsible for the mottled contrast. Evidently, not all of the Al in the reacted region is transformed to d-QC, B2, and Al_9_Ni_2_ forms; some interstitial fcc Al is present as well.

## Discussion

The d-QC most likely formed by melting and mixing of the Al2024 and permalloy followed by rapid solidification. However, simple impedance matching and Hugoniot equation of state calculations show that the temperatures generated by shock compression in the target should not have exceeded 350 °C. Substantially higher temperature is required to melt any of the layers, whether at pressure or upon release to 1 atm. We conclude that additional heating mechanisms must have acted to locally raise the temperature at the interfaces between Al2024 and permalloy 80. As we discuss in Oppenheim *et al*.^10^, the geometry of the experiment is consistent with two distinct heating mechanisms. Collapse of pore spaces created along the interfaces by imperfect machining and frictional sliding due to shear flow across material boundaries with different particle velocities (parallel to the shock propagation direction) both offer plausible local heating mechanisms. The reacted layer studied in detail here is found along a boundary normal to the shock propagation direction and is therefore more likely related to pore collapse.

The d-QC formed in these experiments is very similar to natural decagonite^7^. Although we measure, above detection limit, a number of minor components (Cu, Mg, Mo, and Mn) in the synthetic example, the ratios of the major metals Al_76_Ni_20_Fe_4_ (Table 1) are quite similar to the natural case, Al_71_Ni_24_Fe_5_. Moreover, as we have noted, the layer spacing perpendicular to the 10-fold axis, 4.2 Å, is identical to the more precise value obtained by single-crystal X-ray diffraction on the natural specimen, 4.204(2) Å. Furthermore, like the natural decagonite, our synthetic d-QC shows essentially no measurable phason strain. In the SAED pattern perpendicular to the ten-fold axis (Fig. 5, lower right), we do not observe any weak diffuse reflections, characteristic of imperfectly ordered atoms along the ten-fold axis. Such perfection is surprising given the dynamic synthesis process and the intergrowth with other phases. We made a similar observation in the case of our icosahedral shock-synthetized quasicrystals^9^. In the case of natural decagonite, found in a shocked meteorite billions of years old, Bindi *et al*.^5^ hypothesized that the low phason strain observed might be a side-effect of the original growth process or it might reflect annealing over the very long age of the specimen. In the experimental product, however, such long annealing time has not been available and this supports the notion that shock synthesis grows essentially strain-free highly perfect quasicrystals from the outset.

Al-Ni-Fe decagonal quasicrystals with the composition Al_70_Ni_15_Fe_15_ were first discovered by Tsai *et al*.^17^. By means of a convergent-beam electron diffraction (CBED) study, Saito *et al*.^18^ found so-called G-M lines in odd-order reflections along the c* direction, showing that these quasicrystals belong to the non-centrosymmetric space group *P* $\bar{10}$ *m*2. Their findings were confirmed in follow-up TEM studies by Tsuda *et al*.^19^. Tanaka *et al*.^20^, by CBED and high-resolution microscopy, revealed a transition from *P* $\bar{10}$ *m*2 to the centrosymmetric *P*10_5_/*mmc* as a function of the Ni/Fe ratio. In detail, along the join Al_70_Ni_x_Fe_30-x_ in the range 10 < x < 20, these authors showed that alloys with 10 < x < 17.5 crystallize in the *P* $\bar{10}$ *m*2 structure, whereas alloys with 17.5 < x < 20 give rise to the *P*10_5_/*mmc* structure.

For the decagonal quasicrystal synthesized here, all the TEM-SAEDs we collected that have the c* direction display clear odd-order reflections, indicating the absence of the *c*-glide perpendicular to the 10-fold axis. This likely indicates that the 10_5_ screw axis is absent and the d-QC studied here exhibits the non-centrosymmetric *P* $\bar{10}$ *m*2 structure. Another possibility is that it exhibits a different centrosymmetric space group such as *P*10_5_/*mmm*.

It is noteworthy that the composition of the d-QC reported here is close to that of the thermodynamically stable decagonal phase in the Al-Ni-Fe system, Al_71_Ni_24_Fe_5_^21^, which is known to exhibit the centrosymmetric *P*10_5_/*mmc* structure^22^. The same results were found for the natural analogue of Al_71_Ni_24_Fe_5_, the mineral decagonite^5^, by single-crystal X-ray diffraction^6^. Although it is possible our shock-recovered specimen occupies a metastable or preserved high-pressure structure, we speculate that the discrepancy in the space group could be due to the presence of other minor elements in the chemical formula (i.e., Cu and Mg). Ordering of such minor elements could definitely influence the atomic arrangements sufficiently to make the difference among these rather similar space groups.

In the natural Khatyrka case, decagonite is associated with the bcc-structured steinhardtite phase. However, in this experiment we did not find the bcc structure. Instead we found the stable CsCl-type or B2 structure of AlNiFe, alongside small amounts of interstitial fcc Al and the metastable Al_9_Ni_2_ phase. In fact, studies of various AlNiFe alloys have not uncovered any stability region for steinhardtite either at ambient or elevated pressure^23^. Bindi *et al*.^12^ hypothesized that metastable steinhardtite is formed and recovered in Khatyrka due to the unique properties of shock synthesis, but our experiment provides no support for this particular hypothesis since our experiment instead yielded only B2 AlNiFe alloy alongside the d-QC.

The successful synthesis of decagonal quasicrystals by shock recovery reinforces the conclusion that the so-far unique discovery of natural quasicrystals in the Khatyrka meteorite is explained by a collision experienced by its parent body. Both icosahedral and decagonal quasicrystals can be found together in the same fragments of Khatyrka, e.g., Grain 126,^5,6,12,24^. We have synthesized and recovered these two types of quasicrystals in separate experiments, but the shock conditions achieved in the experiments were nearly identical and well within the range of shock conditions that occur as a shock propagates through a heterogeneous object like a meteorite parent body. Although Khatyrka contains both metallic and silicate components and exothermic reactions between them may have been important in the natural setting, here we successfully observed melting, mixing and quasicrystal formation at interfaces between discrete bulk layers of all-metal starting materials, without any reduction-oxidation reaction. The extraordinary ease with which unmixed metals mix to form quasicrystals under shock stands in stark contrast to conventional metallurgical synthesis methods, which require intimate mixing and controlled quenching of very specific bulk compositions.

## Materials and Methods

### Shock Recovery Experiment

The recovery experiment was constructed as follows. A stainless steel 304 (SS304, Fe_71_Cr_18_Ni_8_Mn_2_Si_1_ with traces of C, S, and P) retainer screw forms a 5 mm thick driver plate at the impact surface. Behind this driver, a 1 mm thick, 4 mm diameter disk of Al2024 alloy (Al_94_Cu_4_Mg_1.5_Mn_0.5_, with no more than 0.5% of any other element) was surrounded laterally by a 1 mm thick, 4 mm inner diameter, 5 mm outer diameter ring of permalloy 80 (Ni_80_Fe_15_Mo_4.5_Mn_0.5_, with less than 0.3% Si) and backed by a 1 mm thick, 5 mm diameter disk of permalloy 80. The permalloy parts were in turn surrounded and backed by a 2 cm thick SS304 inner screw with a 2 mm deep, 5 mm diameter counterbore machined into it to hold the sample. The inner and outer screw were contained in a 3 cm thick, 5 cm diameter SS304 outer housing and momentum trap^25^. Machining imperfections leave various cavities and grooves up to ~5 μm in size at contacts between the various parts; the chamber is pumped to moderate vacuum before shooting but there is low-pressure gas in these void spaces.

The target was impacted by a 2 mm thick Ta flyer carried by a 20 mm diameter plastic sabot. The projectile velocity, measured by dual laser interrupts, was 1041 ± 1 ms^−1^. Given this velocity estimate and the known Hugoniots of Ta, SS304, and Al2024 and an estimate of the permalloy 80 Hugoniot from mixture theory and the shock properties of Ni, Fe and Mo^26–28^, we estimated the states in each material after passage of the first shock with analytical propagation of the uncertainties (Table 2). However, given the thickness of the flyer and the duration of supported shock, there were reverberations within the low-impedance Al2024 layer (Fig. S2). There was time (~0.6 μs) for at least four reverberations, which would have raised the pressure in the Al2024 layer, stepwise, to a pressure very close to the capsule pressure of 26.9 ± 0.1 GPa and an estimated peak temperature ≤350 °C.

**Table 2:** First and peak shock states in experiment S1235.

	SS304	Al2024	Permalloy 80	SS304
P_first_ (GPa)	26.9 ± 0.1	15.5 ± 0.1	22.2 ± 0.1	23.0 ± 0.1
T_first_ (°C)	386	142	116	121
P_peak_ (GPa)	26.9 ± 0.1	26.9 ± 0.1	23.0 ± 0.1	23.0 ± 0.1
T_peak_ (°C)	386	314	235	121

Estimates from successive impedance matches. A 2 mm thick Ta flier launched at 1041 ± 1 ms^−1^ impacted, in sequence, a 5 mm thick SS304 driver, a 1 mm Al2024 disk, a 1 mm thick permalloy 80 disk, and a 2 cm thick SS304 inner screw.

After each shot, the chamber was recovered intact, sawn open along a plane parallel to the shock direction, polished with abrasives down to 0.25 μm and then by 24 hours of vibrational polishing in 30 nm colloidal silica, and examined by scanning electron microscopy, including EDS and EBSD maps and point analyses, by EPMA, and by extracting a thin section by Focused Ion Beam (FIB) milling for TEM analysis.

### Scanning Electron Microscopy

Scanning electron microscopy was carried out with the California Institute of Technology (Caltech) Geological and Planetary Sciences Division (GPS) analytical facility’s Zeiss 1550VP field-emission SEM. Imaging and EDS analyses used 15 kV accelerating potential and a 60 μm beam aperture. EBSD analyses used 20 kV accelerating potential and a 120-μm beam aperture in high-current mode. EDS spectra were on an Oxford X-max Si-Drift Detector. EBSD patterns were collected with an HKL system. Both were analyzed using the Oxford Instruments AZtec software.

### Electron Probe

Selected areas of the recovered samples were reanalyzed for Al, Cu, Fe, Cr, Ni, Mo, Mn, Mg, and Si with the five spectrometer JEOL 8200 electron microprobe in Caltech’s GPS analytical facility, using 12 kV accelerating potential, a focused 5 nA beam, 20 s counting times on peak and 10 s on each background position, and pure metal standards.

### Transmission Electron Microscopy

The FIB and TEM facilities used are in the Kavli Nanoscience Institute at Caltech. FIB section A was milled and lifted out from quasicrystal-bearing regions using an FEI Nova 600 Nanolab DualBeam FIB and SEM using a 30 kV Ga-ion beam for initial milling. After placement on a copper TEM grid, this sample was thinned and finalized with an 8 kV 19 nA Ga-ion beam. FIB section B was thinned to 700 nm on the Nova 600 with 30 kV Ga beam and then transferred to a. Zeiss Orion NanoFab to finalize the sample thinning to ~100 nm, with a 5 kV Ga beam. Analytical transmission electron microscopy (ATEM) analysis was performed on a FEI Tecnai TF20 instrument with super-twin objective lenses, operated at 200 kV. The energy dispersive spectroscopy (EDS) data were collected in TEM mode using an EDAX SiLi detector with 10 eV/channel and 51.2 µs processing time, to achieve 500 counts per second signals and 20–50% dead time. The SEAD patterns were integrated using Gatan DigitalMicrograph™ to refine the *d*-spacings of the studied quasicrystals.

### Data and materials availability

The recovered specimen is archived in the Lindhurst Laboratory at Caltech and requests for further study may be directed to the first author. Original images and spectra are archived in the Caltech GPS Division Analytical Facility and Kavli NanoScience Institute and are available upon request.

## Electronic supplementary material

Supplementary Materials
