# Supplementary material for: Shock Synthesis of Decagonal Quasicrystals
Source: Sci Rep. 2017 Nov 15;7:15628. doi: 10.1038/s41598-017-15229-4 (PMC5688080; doi:10.1038/s41598-017-15229-4)
Supplement: Supplementary file 1 — Supplementary Materials [file 41598_2017_15229_MOESM1_ESM.pdf]

Supplementary Materials for Shock Synthesis of Decagonal Quasicrystals

J. Oppenheim[1], C. Ma[1], J. Hu[1], L. Bindi[2,3], P. J. Steinhardt[4,5], and P. D. Asimow[1]*

[1]Division of Geological and Planetary Sciences, California Institute of Technology, 1200 E. California Blvd. M/C170-25, Pasadena, CA 91125, U.S.A.

[2]Dipartimento di Scienze della Terra, Università degli Studi di Firenze, Via La Pira 4, I-50121 Firenze, Italy

[3]CNR-Istituto di Geoscienze e Georisorse, Sezione di Firenze, Via La Pira 4, I-50121 Firenze, Italy

[4]Department of Physics, Princeton University, Jadwin Hall, Princeton, NJ 08544, U.S.A.

[5]Princeton Center for Theoretical Science, Princeton University, Princeton, NJ 08544, U.S.A.

** Corresponding author: Asimow@gps.caltech.edu, +1(626)395-4133, FAX +1(626)395-1995*

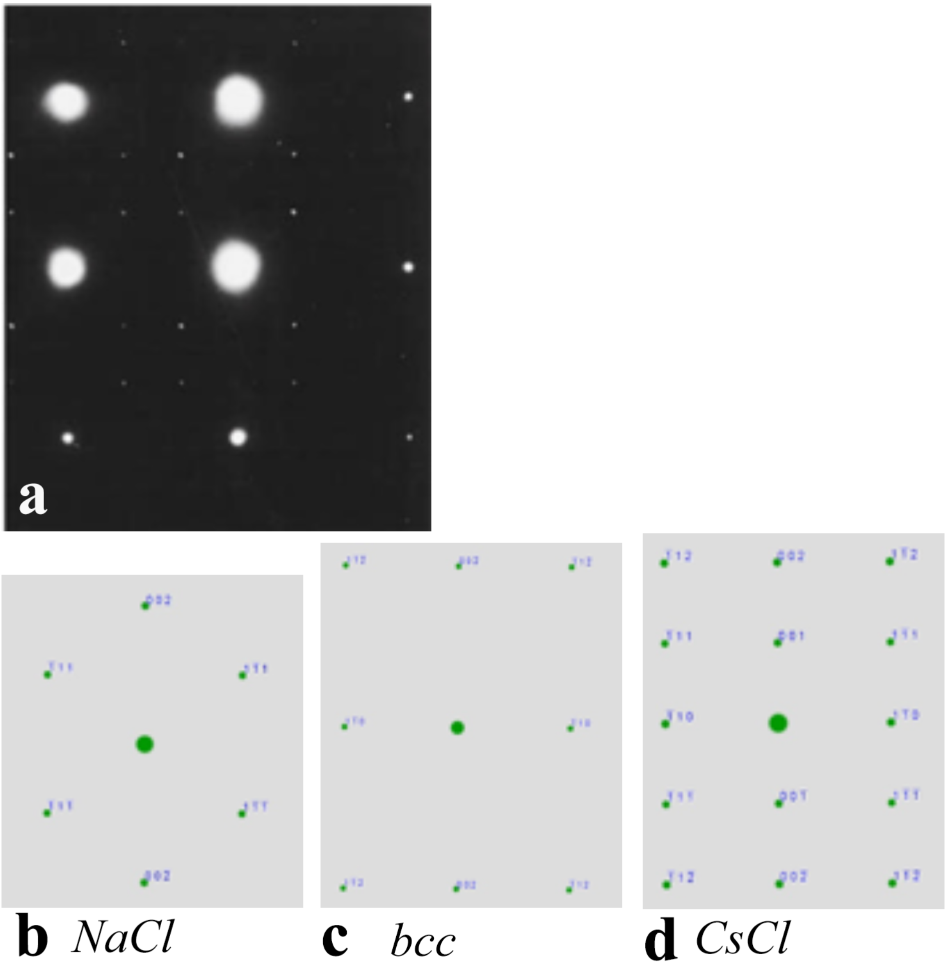

Fig. S1. Example diffraction patterns to distinguish space groups and recognize superlattices. Left: Superlattice diffraction pattern of a non-stoichiometric V_6C_5 ceramic [14]. The pattern is comparable to the superlattice diffraction of the Al-alloy in Figs. 6 and 7. Middle: the [110] zone diffraction pattern of NaCl-type face-centered cubic structure (top) and of Steinhardtite-type body-centered cubic structure (bottom). Right: the [110] zone diffraction pattern of CsCl-type primitive lattice, which most closely matches the Al-alloy in Fig. 6.

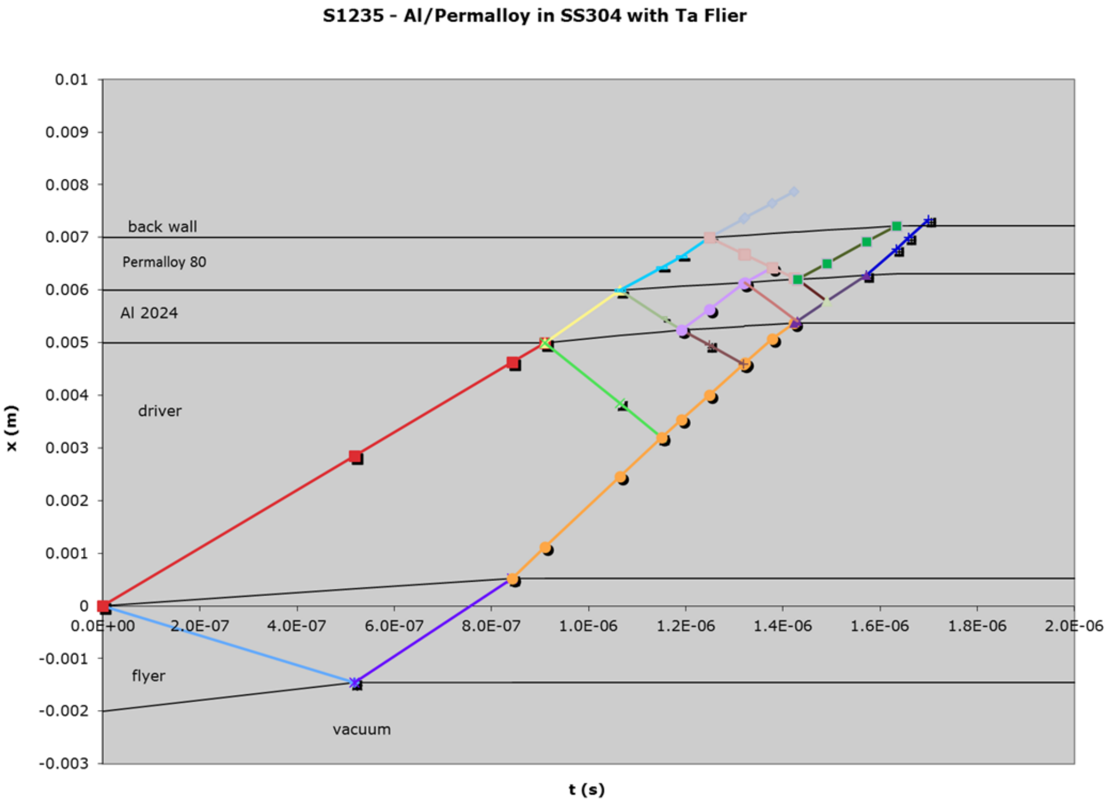

Fig. S2. Distance-time plot showing estimated wave propagation and interactions in shot S1235. The Al2024 layer experienced four shocks (two from the SS304 driver and two from the permalloy 80 layer behind it) before the release wave overtook the sample chamber. The Al2024-permalloy interface was at high pressure for about 0.6 μs. Key: Red squares = first shock in driver; cyan diamonds = first shock in flyer; purple asterisks = release in flyer; orange circles = forward-going release in driver; light green x's = rear-going partial release in driver; yellow triangles = first shock in Al2024; olive dashes = second shock in Al2024; lavender circles = third shock in Al2024; brown pluses = second shock in driver; ochre = third shock in Al2024; sky blue em-dashes = first shock in permalloy 80; dark purple triangles = release in Al2024; dark blue pluses = release in permalloy 80. Additional reflections shown in permalloy 80 layer are negligible due to small impedance contrast with back wall of 304SS.
